# West Nile Virus Infection and Conjunctival Exposure

**DOI:** 10.3201/eid1110.040212

**Published:** 2005-10

**Authors:** Kevin Fonseca, Gerry D. Prince, Jeff Bratvold, Julie D. Fox, Margo Pybus, Jutta K. Preksaitis, Peter Tilley

**Affiliations:** * Provincial Laboratory for Public Health (Microbiology), Calgary, Alberta, Canada; † Medicine Hat, Alberta, Canada; ‡ Alberta Fish & Wildlife, Edmonton, Alberta, Canada

**Keywords:** West Nile virus, occupational exposure, conjunctival exposure, flavivirus, corvid, crow, transmission, letter

**To the Editor:** Corvids (crows, blue jays, magpies, and their relatives) are particularly susceptible to West Nile virus (WNV) (*1*). Birds are useful indicators of the spread of WNV (*1*), and Canada has implemented WNV surveillance strategies that use these species as sentinels.

Direct acquisition of WNV through percutaneous injuries has been reported in 2 laboratory circumstances, involving a blue jay and a mouse (*2*). We describe a conjunctival exposure to WNV that occurred in the field and probably resulted in infection in the exposed person.

As part of the local WNV bird surveillance activities in 2003, an animal control officer collected sick and dead corvids at the Canadian Forces Base, Suffield, Alberta. He had a protective suit on, but he wore no mask or face shield. While killing an injured crow (*Corvus brachyrhynchos*), the officer struck the struggling bird on a nearby horizontal pipe gate, which resulted in fracture of the skull, causing brain tissue and cerebrospinal fluid to spray onto his head, face, neck, and right shoulder. Body fluids and brain material of the bird entered his eyes, but not his mouth; he had no known open lesions on the exposed area. His co-workers immediately wiped off visible material, and a few hours later he showered.

The dead crow was sent for analysis to the Fish and Wildlife Division, Government of Alberta, where laboratory tests using the VecTest assay (Medical Analysis Systems, Inc., Camarillo, CA, USA), indicated that the crow was positive for WNV antigen. This test has been validated for detecting viral antigen in oropharyngeal and cloacal swabs in crows (*3*).

Seven days after exposure, the animal control officer became unwell and sought medical assistance. His symptoms included headaches, dizziness, spiking fevers, and sweats; on examination, mild otitis was noted, but he did not display meningismus or other neurologic signs. A whole blood sample with EDTA and a serum sample were collected, together with a throat swab for viral culture to exclude a possible enteroviral infection, as part of a standardized provincial protocol for investigating suspected WNV infections in Alberta. Betahistine dihydrochloride was prescribed for the dizziness and a cephalosporin for otitis. A cerebrospinal fluid sample was not collected, since his clinical signs did not suggest neurologic involvement.

At the Provincial Laboratory, WNV RNA was detected in the plasma by nucleic acid sequence-based amplification, with primers described by Lanciotti and Kerst (*4*), which was confirmed by the Artus RealArt RT-PCR assay (artus biotech USA Inc, San Francisco, CA, USA) in a Roche LightCycler. The serum sample, collected at the same time as the plasma sample, was negative for immunoglobulin M (IgM) antibody by enzyme immunoassay using 2 kits (Panbio, Windsor, Queensland, Australia; and Focus Technologies, Cypress, CA, USA). Fourteen days after exposure, a convalescent-phase serum sample showed IgM antibody to WNV in both kits; the plasma sample was negative for viral RNA. Hemagglutination inhibition assay on the acute- and convalescent-phase serum samples, collected 7 days apart, showed rising titers, from <1:10 on the acute-phase serum, to 1:40 for dengue virus (serotypes 1–4), 1:40 for St. Louis encephalitis virus, and 1:80 for WNV on the convalescent-phase serum. Preliminary data from our laboratory indicate that in ≈40% of cases of acute West Nile fever, the acute-phase plasma sample shows viral RNA before IgM antibody develops, after which viral RNA is no longer detectable (J. Fox, unpub. data). Two weeks after culture was initiated for virus isolation, the throat swab was negative for enteroviruses.

The patient's severe fever, sweats, headaches, anorexia, fatigue, and diminished concentration and memory continued. His symptoms peaked 2 weeks after the initial exposure. Three months later, his symptoms of fatigue, dizziness, headaches, and poor memory were severe enough to prevent him from returning to fulltime work. Eight months after exposure, he continues to have fatigue and headaches.

We believe this is the first reported case of apparent conjunctival transmission of WNV in an occupational setting. As the officer spent considerable time outdoors in areas where WNV transmission was relatively high in 2003 and repeatedly handled infected birds, we cannot eliminate the possibility of a mosquito bite or other percutaneous route of transmission. However, the nature of the exposure and the time to symptom development strongly suggest that infection occurred after conjunctival exposure. Persons who dispatch sick wildlife are encouraged to use appropriate, humane methods and should take precautions against exposure to tissues and body fluids.
